# Advances in natural coumaronochromones: a comprehensive review of natural occurrence, bioactivities, and chemical synthesis

**DOI:** 10.1007/s13659-026-00627-x

**Published:** 2026-04-01

**Authors:** Dan Xia, Qin Lv, Wei Wang, Meng-Fan Xie, Jin-Cai Li, Kun Li, Dashan Li, Wen-Jing Wang, Li-Dong Shao

**Affiliations:** 1 Yunnan Key Laboratory of Southern Medicinal Utilization, School of Chinese Materia Medica, Yunnan University of Chinese Medicine, Kunming, 650500 China; 2 School of Basic Medical Sciences, Yunnan University of Chinese Medicine, Kunming, 650500 China

**Keywords:** Coumaronochromones, Natural occurrence, Biological activities, Chemical syntheses

## Abstract

Coumaronochromones, characterized by a distinctive benzofuro[2,3-*b*]chromenone core, constitute a rare subclass of isoflavonoids. Biosynthetically, they are proposed to originate from carbohydrate metabolism and the phenylpropanoid pathway like other isoflavones. To date, 92 natural coumaronochromones have been isolated and characterized from nearly 28 species across 24 genera within 7 families (including one fungal source). These compounds are predominantly distributed in the Fabaceae family, which accounts for over half (64%) of all known derivatives. Notably, approximately 20% of coumaronochromones are derived from the genus *Euchresta* (Fabaceae). The unique scaffold of coumaronochromones confers a broad spectrum of notable pharmacological activities, including anti-inflammatory, antibacterial, insecticidal, cytotoxic, and immunomodulatory effects, highlighting their potential as valuable natural leads for pharmaceutical and agrochemical development. This review comprehensively summarizes recent advances in the chemistry and biology of coumaronochromones, providing a detailed account of their natural occurrence, documented biological activities, and chemical syntheses.

**Graphical Abstract:**

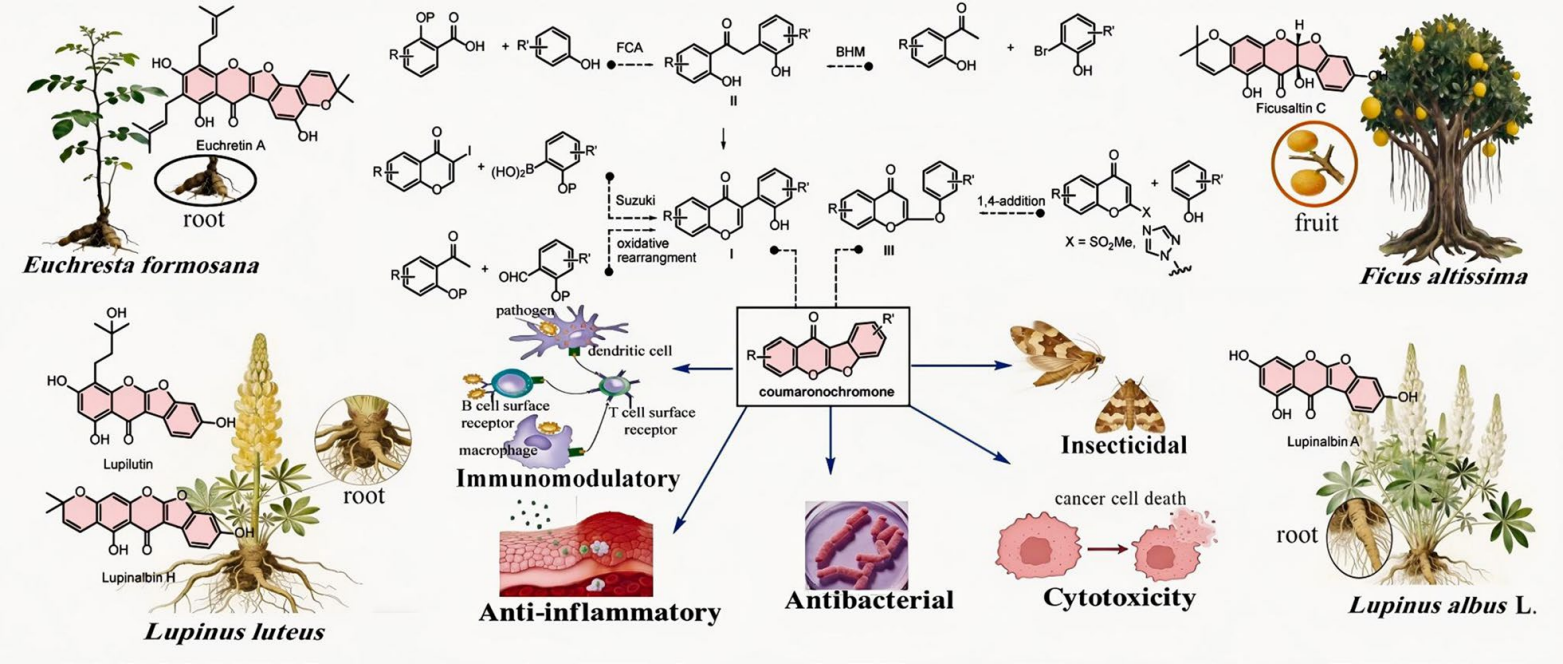

## Introduction

**Scheme 1:**
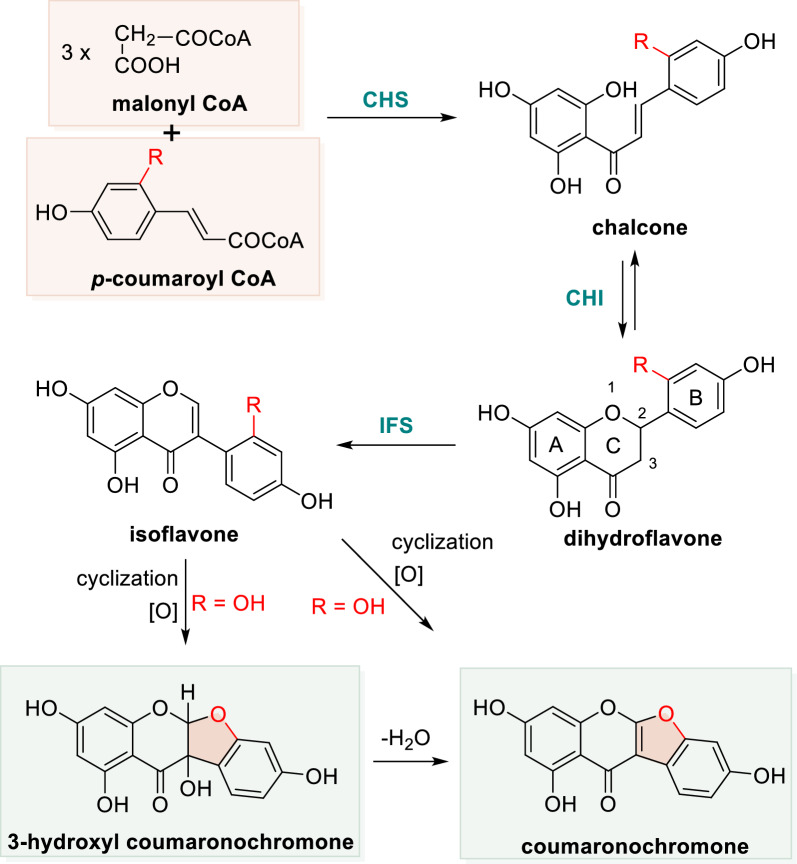
Plausible biosynthetic pathways for coumaronochromone

Coumaronochromones represent a rare class of isoflavonoid natural products characterized by a distinctive benzofuro[2,3-*b*]chromenone core (Scheme 1). Primarily found in leguminous plants, they exhibit a broad spectrum of significant physiological and pharmacological activities. Reported biological properties include anti-inflammatory, antibacterial, antitumor, insecticidal, and immunomodulatory effects, highlighting their considerable therapeutic and agrochemical potential [1–9]. To date, 92 natural coumaronochromones have been identified from approximately 28 species spanning 24 genera across 7 families (including one fungal source). Despite their diverse bioactivities and promising applications—ranging from pharmaceuticals to food additives and agricultural agents—a comprehensive review systematically covering their botanical origins, biological functions, synthetic methodologies, and practical applications remains unavailable. This review aims to address this gap by consolidating recent advances in the chemistry and biology of coumaronochromones. It provides a detailed overview of their natural occurrence (plant sources), documented biological activities, and reported chemical syntheses. By organizing existing knowledge and highlighting future directions, this work is intended to serve as a valuable resource for researchers in natural products chemistry, organic synthesis, and medicinal chemistry.

Coumaronochromones are classified as a subclass of isoflavones, which are plant-derived secondary metabolites. Isoflavones represent a major group of flavonoids, structurally characterized by the migration of the B-ring from the C-2 to the C-3 position of the flavonoid core. While the complete biosynthetic pathway of coumaronochromones remains unresolved, it is hypothesized that their upstream biosynthesis aligns with that of isoflavones (Scheme 1). The biosynthesis of isoflavones in plants is well-established, initiating from malonyl-CoA and *p*-coumaroyl-CoA—precursors derived from carbohydrate metabolism and the phenylpropanoid pathway, respectively. The core scaffold is assembled from three malonyl-CoA molecules and one coumaroyl-CoA molecule through sequential enzymatic reactions. First, chalcone synthase (CHS) catalyzes the condensation of these substrates to form a chalcone intermediate. This chalcone is subsequently isomerized by chalcone isomerase (CHI) to yield a dihydroflavone. As documented in the literature, dihydroflavone is then converted into the corresponding isoflavone by isoflavone synthase (IFS) [10, 11].

Based on this pathway, we propose a biosynthetic hypothesis wherein isoflavones bearing a hydroxyl group at a specific position (when R=OH) may undergo further cyclization and oxidation to generate coumaronochromones and 3-hydroxycoumaronochromones. Whether this hydroxyl group originates from the initial substrate or is introduced via a later oxidative step remains to be clarified through detailed biosynthetic studies.

## Plant sources

To date, a total of 92 coumaronochromones have been isolated and characterized from natural sources, encompassing 28 species distributed across 24 genera within 7 families (including one fungal species). The structures, botanical origins, and plant parts of these compounds are summarized in Table 1, with further contextual details provided in the accompanying text.

**Table 1:** The plant sources of natural coumaronochromones

Structure	Name	Plants	Parts	Bioactivity	References
	Lupinalbin A (**1**)	*luteus* *L. albus* *A. americana*	Roots/rhizomes	–	[12–14]
	Lupinalbin B (**2**)	*luteus* *L. albus* *F. altissima*	Roots/fruits	–	[12, 18, 54]
	Lupinalbin C (**3**)	*L. albus* L	Roots	–	[12]
	Lupinalbin D (**4**)	*L. albus* L	Roots	–	[12]
	Lupinalbin E (**5**)	*L. albus* L	Roots	–	[12]
	Lupinalbin G (**6**)	*L. albus* L. *L. albus*	Roots	–	[12, 13]
	Lupinalbin F (**7**)	*L. luteus*	Roots	–	[16]
	Lupinalbin H (**8**)	*L. luteus*	Roots	–	[17]
	Lupilutin (**9**)	*L. luteus*	Roots	–	[19]
	Lupalbin B (**10**)	*L. luteus*	Roots	–	[19]
	Euchretin A (**11**)	*japonica* *E. formosana*	Stems/roots	Cytotoxicity	[20, 21]
	Euchretin D (**12**)	*E. formosana*	Roots	–	[22]
	Euchretin E (**13**)	*E. formosana*	Roots	–	[22]
	Euchretin G (**14**)	*E. japonica*	Roots	–	[23]
	Euchretin J (**15**)	*E. formosana*	Roots	Cytotoxicity	[21]
	Euchretin K (**16**)	*E. formosana*	Root	–	[21]
	Euchretin L (**17**)	*E. formosana*	Roots	–	[21]
	Euchretin M (**18**)	*E. formosana*	Roots	Cytotoxicity	[21]
	Euchretin N (**19**)	*E. formosana*	Roots	–	[21]
	Formosanatin A (**20**)	*E. formosana*	Roots	–	[22]
	Formosanatin B (**21**)	*E. formosana*	Roots	–	[22]
	Formosanatin C (**22**)	*E. formosana*	Roots	–	[22]
	Formosanatin D (**23**)	*E. formosana*	Roots	–	[22]
	Euchretin B (**24**)	*E. formosana*	Stems	–	[24]
	Euchretin C (**25**)	*E. formosana*	Stems	–	[24]
	Euchretin F (**26**)	*E. japonica*	Roots	–	[23]
	Euchretin H (**27**)	*E. japonica*	Roots	–	[23]
	Euchretin I (**28**)	*E. tubulosa*	Roots	Anti-inflammatory	[25, 36]
	Desmoxyphyllin A (**29**)	*D. oxyphyllum*	Leaves	–	[26]
	Desmoxyphyllin A 7-*O*-*β*-D-glucopyranoside (**30**)	*D. oxyphyllum*	Leaves	–	[26]
	Desmoxyphyllin B (**31**)	*D. oxyphyllum*	Leaves	–	[26]
	Desmoxyphyllin B 7-*O*-*β*-D-glucopyranoside (**32**)	*D. oxyphyllum*	Leaves	–	[26]
	Lisetinone (**33**)	*P. erythrina*	Root barks	–	[28]
	Lisetm hydrate (**34**)	*P. erythrina*	Root barks	–	[28]
	Lisetin (**35**)	*P. erythrina*	Root barks	–	[28]
	8-prenyl-lisetin (**36**)	*P. erythrina*	Root barks	–	[28]
	Piscerythrol (**37**)	*P. erythrina*	Root barks	–	[28, 29]
	Obiongin (**38**)	*D. elliptica* *D. oblonga*	Aerial/roots	Insecticidal	[30, 32]
	Oblonginol (**39**)	*D. oblonga*	Roots	–	[30]
	Erysenegalensein J (**40**)	*E. seneoalensis*	Stem barks	–	[31]
	6,4'-dihydroxy-7,5'-dimethoxycoumaronochromone (**41**)	*D. elliptica*	Aerial parts	Insecticidal	[32]
	Erythrinasubumbrin A (**42**)	*E. subumbrans*	Twigs/leaves	Anti-inflammatory	[33]
	(+)-(2*S*,3*R*)-Erythrinasubumbrin B (**43**)	*E. subumbrans*	Twigs/leaves	Anti-inflammatory	[33]
	(−)-(2*R*,3*S*)-Erythrinasubumbrin B (**44**)	*E. subumbrans*	Twigs/leaves	Anti-inflammatory	[33]
	Lupinol C (**45**)	*E. subumbrans*	Twigs/leaves	Anti-inflammatory	[33]
	3,5,7,4'-tetrahydroxy-coumaronochromone (**46**)	*D. styracifolium*	Aerial	–	[34]
	Nor-dehydrodeguelin (**47**)	*L. nicou*	Roots	–	[35]
	(-)-nor-dehydrorotenone (**48**)	*L. nicou*	Roots	–	[35]
	Hirtellanine A (**49**)	*C. hirtella*	Roots	Immunomodulatory	[8]
	4',5'-dihydroxy-5,7-dimethoxy-6-(3-methylbut-2-enyl)coumaronochromone (**50**)	*C. hirtella*	Roots	Immunomodulatory	[36, 37]
	3,8,9,16-tetrahydroxy-18-methoxy-2-((*R*)-5-methoxy-2,2-dimethyl-7,8-dihydro-2*H*,6*H*-pyrano[3,2-*g*]chromen-7-yl)-17-(3-methylbut-2-en-1-yl)-11*H*-10*b*,5*a*-(epoxy[1,2]benzeno)benzofuro[2,3-*b*]chromen-11-one (**51**)	*C. hirtella*	Roots	–	[37]
	Sophorophenolone (**52**)	*S. japonica*	Pericarps	–	[38]
	Dalbergichromone (**53**)	*D. boehmii*	Leaves/heartwoods	–	[3]
	Eriocoumaronochromone (**54**)	*E. robustum*	Twigs	Antibacterial	[2]
	(2*R*,3*S*)-3,7,4'-trihydroxy-5-methoxycoumaronochromone (**55**)	*A. americana*	Rhizomes	Anti-inflammatory	[15]
	7,3'-dihydroxy-2',4'-dimethoxycoumaronochromone (**56**)	*M. lasiantha*	Roots	–	[39]
	Cajasan (**57**)	*C. cajan* L	Cell culture	Anti-inflammatory	[40]
	Ayamenin A (**58**)	*I. pseudacorus*	Leaves	–	[41]
	Ayamenin B (**59**)	*I. pseudacorus*	Leaves	–	[41]
	Ayamenin C (**60**)	*I. pseudacorus*	Leaves	–	[41]
	Ayamenin D (**61**)	*I. pseudacorus*	Leaves	–	[41]
	5,7,3'-trihydroxy-6-methoxycoumaronochromone (**62**)	*I. pseudacorus*	Leaves	–	[41]
	Irisbungin (**63**)	*I. bungei*	Leaves	–	[42]
	Urophyllumol (**64**)	*U. chinensis*	Branche/leaves	–	[43]
	Boeravinone J (**65**)	*B. diffusa*	Roots	–	[44]
	Boeravinone R (**66**)	*B. diffusa*	Roots	–	[45]
	Boeravinone Y (**67**)	*A. nana*	Cell culture	Anti-inflammatory	[7]
	Aervin A (**68**)	*A. persica*	Whole plant	Antibacterial	[46, 47]
	Aervin B (**69**)	*A. persica*	Whole plant	Antibacterial	[46, 47]
	Aervin C (**70**)	*A. persica*	Whole plant	Antibacterial	[46, 47]
	Cristatone I (**71**)	*C. cristata*	Inflorescences	–	[48, 49]
	Cristatone II (**72**)	*C. cristata*	Inflorescences	Cytotoxicity	[6, 48, 49]
	Suaeglaucin A (**73**)	*S. glauca*	Whole plant	–	[50]
	Agrisquarin D (**74**)	*A. squarrosum* L	Whole plant	Anti-inflammatory	[51]
	Agrisquarin E (**75**)	*A. squarrosum* L	Whole plant	Anti-inflammatory	[51]
	( ±)-Agrisquarin G (**76**)	*A. squarrosum* L	Whole plant	Anti-inflammatory	[51]
	Suaeglaucin C (**77**)	*S. glauca*	Rhizomes	Cytotoxicity	[52]
	Baeckein F (**78**)	*B. frutescens* L	Roots	Anti-inflammatory	[9]
	Baeckein G (**79**)	*B. frutescens* L	Roots	Anti-inflammatory	[9]
	Baeckein H (**80**) R = *β*-D-glu	*B. frutescens* L	Roots	Anti-inflammatory	[9]
	Baeckein I (**81**) R = *β*-D-glu	*B. frutescens* L	Roots	Anti-inflammatory	[9]
	Lotuschromone (**82**)	*mycorrhiza*	Cell culture	–	[15]
	Ficusaltin C (**83**)	*F. altissima*	Fruits	–	[18, 53]
	Ficusaltin D **(84**)	*F. altissima*	Fruits	Anti-inflammatory	[18, 53]
	Ficusaltin E (**85**)	*F. altissima*	Fruits	–	[53]
	Ficusaltin F (**86**)	*F. altissima*	Fruits	–	[53]
	(2*R*,3*S*)-3,5,7-trihydroxy-4'-methoxycoumaronochromone (**87**)	*F. altissima*	Fruits	–	[53]
	(2*S*,3*R*)-3,5,7-trihydroxy-4'-methoxycoumaronochromone (**88**)	*F. altissima*	Fruits	–	[53]
	(2*S*,3*R*)-6-(3-methyl-2-buten-1-yl)-3,5,7-trihydroxy-4'-methoxycoumaranochroman-4-one) (**89**)	*F. altissima*	Fruits	–	[18]
	(2*R*,3*S*)-6-(3-methyl-2-buten-1-yl)-3,5,7-trihydroxy-4'-methoxycoumaranochroman-4-one (**90**)	*F. altissima*	Fruits	–	[18]
	(2*S*,3*R*)-6-(3-methyl-2-buten-1-yl)-3,5,7,4'-tetrahydroxy-coumaranochroman-4-one (**91**)	*F. altissima*	Fruits	–	[18]
	(2*R*,3*S*)-6-(3-methyl-2-buten-1-yl)-3,5,7,4'-tetrahydroxy-coumaranochroman-4-one (**92**)	*F. altissima*	Fruits	–	[18]

Analysis of their distribution indicates that coumaronochromones occur predominantly within the Fabaceae family, from which more than half (64%) of all known derivatives have been isolated. The Moraceae family represents the second most significant source, accounting for 14% of reported compounds. Notably, approximately 20% of all coumaronochromones have been identified from plants belonging to the genus *Euchresta* (Fabaceae). Among these species, *Euchresta japonica* has a long history of use in traditional Chinese medicine (TCM), where it is employed for its heat-clearing, detoxifying, throat-soothing, and anti-inflammatory properties.

### Fabaceae

Roots of *Lupinus albus* L.: lupinalbins A–E (**1–5**), and lupinalbin G (**6**) [12, 13].

Roots of *Lupinus luteus*: lupinalbins A and B (**1** and **2**) [12, 14, 15], lupinalbin D (**4**) [12], lupinalbin F (**7**) [12, 16], lupinalbin H (**8**) [17], lupilutin (**9**), and lupalbin B (**10**) [12, 18, 19].

Roots of *Euchresta formosana*: euchretin A (**11**) [20, 21], euchretins D and E (**12** and **13**) [22], euchretin G (**14**) [23], euchretins J-N (**15–19**), formosanatins A-D (**20–23**) [22].

Stems of *Euchresta formosana*: euchretins B and C (**24** and **25**) [24].

Roots of *Euchresta japonica*: euchretin G (**14**), euchretins F and H (**26** and **27**)[23].

Roots of *Euchresta tubulosa*: euchretin I (**28**) [25].

Leaves of *Desmodium oxyphyllum*: desmoxyphyllin A (**29**), desmoxyphyllin A 7-*O*-*β*-D-glucopyranoside (**30**), desmoxyphyllin B (**31**), desmoxyphyllin B 7-*O*-*β*-D-glucopyranoside (**32**) [26].

Root barks of *Piscidia erythrina*: lisetinone (**33**), lisetm hydrate (**34**), lisetin (**35**), 8-prenyl-lisetin (**36**), ptscerythro (**37**) [27–29].

Roots of *Derris oblonga*: obiongin (**38**) and oblonginol (**39**) [30].

Stem barks of *Erythrina seneoalensis*: erysenegalensein J (**40**) [31].

Arial parts of *Derris elliptica*: obiongin (**38**) and 6,4'-dihydroxy-7,5'-dimethoxy-coumaronochromone (**41**) [30, 32].

Twigs and leaves of *Erythrina subumbrans*: erythrinasubumbrin A (**42**) and (+)-erythrinasubumbrin B (**43**), (−)-erythrinasubumbrin B (**44**), and lupinol C (**45**)[33].

Arial parts of *Desmodium styracifolium*: 3,5,7,4'-tetrahydroxycoumaronochromone (**46**) [34].

Roots of *Lonchocarpus nicou*: *nor*-dehydrodeguelin (**47**) and (-)-nor-dehydrorotenone (**48**) [35].

Roots of *Campylotropis hirtella*: hirtellanine A (**49**) [8], 4',5'-dihydroxy-5,7-dimethoxy-6-(3-methylbut-2-enyl)coumaronochromone (**50**) [36] and 3,8,9,16-tetrahydroxy-18-methoxy-2-((*R*)-5-methoxy-2,2-dimethyl-7,8-dihydro-2*H*,6*H*-pyrano[3,2-*g*]chromen-7-yl)-17-(3-methylbut-2-en-1-yl)-11*H*-10b,5a-(epoxy[1,2]benzeno)benzofuro[2,3-*b*]chromen-11-one (**51**) [37].

Pericarps of *Sophora japonica*: sophorophenolone (**52**) [38].

Leaves and heartwood of *Dalbergia boehmii*: dalbergichromone (**53**) [3].

Twigs of *Eriosema robustum*: eriocoumaronochromone (**54**) [2].

Rhizomes of *Apios americana*: (2*R*,3*S*)-3,7,4'-trihydroxy-5-methoxycoumaronochromone (**55**) [15].

Roots of *Millettia lasiantha*: 7,3'-dihydroxy-2',4'-dimethoxycoumaronochromone (**56**) [39].

Roots of *Cajanus cajan* L.: cajasan (**57**) [40].

### Iridaceae

Leaves of *Iris pseudacorus*: ayamenins A–D (**58–61**), 5,7,3'-trihydroxy-6-methoxycoumaronochromone (**62**) [41].

Leaves of *Iris bungei* Maxim.: irisbungin (**63**) [42].

### Rubiaceae

Twigs and leaves of *Urophyllum chinense* Merr. & Chun: urophyllumol (**64**) [43].

### Nyctaginaceae

Roots of *Boerhaavia diffusa*: boeravinone J (**65**) [44] and boeravinone R (**66**) [45].

Cell culture of *Abronia nana*: boeravinone Y (**67**) [7].

### Amaranthaceae

Whole plant of *Aerva persica*: aervins A–C (**68–70**) [46, 47].

Inflorescences of *Celosia cristata*: cristatones I and II (**71** and **72**) [48, 49].

Whole plant of *Suaeda glauca*: suaeglaucin A (**73**) [50].

Whole plant of *Agriophyllum squarrosum*: agrisquarins D and E (**74** and **75**), and (±)-agrisquarin G (**76**) [51].

Rhizomes of *Suaeda glauca*: suaeglaucin C (**77**) [52].

### Myrtaceae

Roots of *Baeckea frutescens* L.: baeckeins F–I (**78–81**) [9].

### Arbuscular mycorrhizal fungi

Cell culture of *Arbuscular mycorrhiza*: lotuschromone (**82**) [15].

### Moraceae

Fruits of *Ficus altissima*: lupinalbin B (**2**), lupinalbin D (**3**), ficusaltins C–F (**83–86**), (2*R*,3*S*)-3,5,7-trihydroxy-4'-methoxycoumaronochromone (**87**), (2*S*,3*R*)-3,5,7-trihydroxy-4'-methoxycoumaronochromone (**88**), (2*S*,3*R*)-6-(3-methyl-2-buten-1-yl)-3,5,7-trihydroxy-4'-methoxycoumaranochroman-4-one) (**89**), (2*R*,3*S*)-6-(3-methyl-2-buten-1-yl)-3,5,7-trihydroxy-4'-methoxycoumaranochroman-4-one (**90**), (2*S*,3*R*)-6-(3-methyl-2-buten-1-yl)-3,5,7,4'-tetrahydroxycoumaranochroman-4-one (**91**), and (2*R*,3*S*)-6-(3-methyl-2-buten-1-yl)-3,5,7,4'-tetrahydroxycoumaranochroman-4-one (**92**) [18, 53, 54].

## Bioactivities

**Fig. 1:**
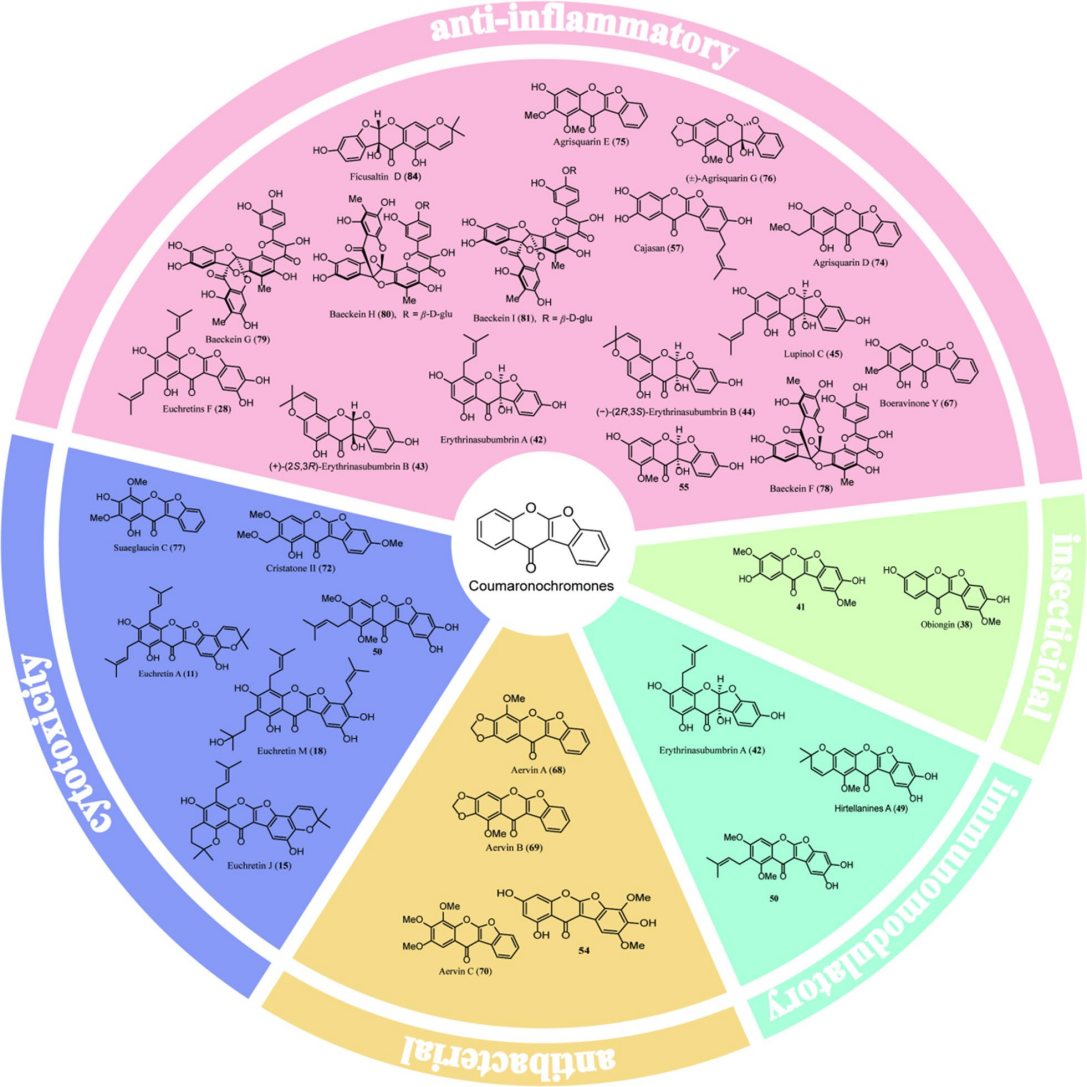
An overview of biological activities of natural coumaronochromones

Despite no coumaronochromone-derived drugs having reached clinical development or the market, natural coumaronochromones exhibit diverse biological activities, notably including anti-inflammatory, antibacterial, cytotoxicity, insecticidal, and immunomodulatory properties (Fig. 1). These findings underscore the potential of coumaronochromones as a valuable source of bioactive leads worthy of further investigation.

### Anti-inflammatory

Euchretin F (**28**) demonstrated significant inhibitory activity against arachidonic acid (AA)- and collagen-induced platelet aggregation [36].

Erythrinasubumbrin A (**42**), (+)-erythrinasubumbrin B (**43**), (-)-erythrinasubumbrin B (**44**), and lupinol C (**45**) significantly suppressed NO production at 10 μM in LPS-stimulated BV-2 cells, with inhibition rates of 37.50%, 24.76%, 40.92%, and 86.17%, respectively (*p* < 0.05) [33].

(2*R*,3*S*)-3,7,4'-Trihydroxy-5-methoxycoumaronochromone (**55**) showed marked anti-inflammatory activity in vitro, effectively inhibiting LPS-induced NO production in RAW 264.7 macrophages with an IC_50_ of 0.38 ± 0.04 μM, suggesting its potential to mitigate inflammatory responses via suppression of excessive NO generation [15].

Boeravinone Y (**67**) was found to effectively inhibit HMGB1-mediated septic responses induced by LPS. In a cecal ligation and puncture (CLP)-induced sepsis model, this compound enhanced macrophage phagocytic activity and promoted bacterial clearance in both peritoneal fluid and blood of septic mice [7].

The anti-inflammatory activities of baeckeins F–I (**78–81**) were also examined in LPS-stimulated RAW 264.7 cells. Following MTT viability testing, baeckein I (**81**) displayed the highest NO-inhibitory activity, with an IC_50_ of 15.2 μM, comparable to that of the positive control indomethacin (IC_50_ = 13.8 μM) [9].

Ficusaltin D (**84**) exhibited notable anti-neuroinflammatory effects. It significantly suppressed LPS-induced NO production in BV-2 cells and down-regulated the expression of iNOS, IL-6, and IL-1β. Furthermore, the compound inhibited nuclear translocation of NF-κB, indicating a multi-target mechanism underlying its anti-neuroinflammatory activity [53].

Cajasan (**57**), agrisquarin D (**74**), agrisquarin E (**75**), and (±)-agrisquarin G (**76**), exhibited certain inhibitory rates of 51.99 ± 4.26%, 5.77 ± 1.24%, 26.54 ± 1.80%, and 16.10 ± 1.04%, respectively, in inhibition of LPS-induced NO production in RAW 264.7 cells (with L-NMMA as a positive control, 52.01 ± 1.96% inhibition) [40, 51].

### Antibacterial

Compound **54** exhibited weak antimicrobial activity, with minimum inhibitory concentrations (MICs) > 150 μg/mL against two Gram-positive bacteria (*Bacillus subtilis* and *Staphylococcus aureus*), two Gram-negative bacteria (*Klebsiella pneumoniae* and *Escherichia coli*), one fungus (*Candida albicans*), and one yeast (*Saccharomyces cerevisiae*)[2].

Imran et al. found aervins A–C (**68–70**) exhibited notable antibacterial activity against a panel of bacterial strains, with MIC values ranging from 60.05 to 79.21 μg/mL. In addition, compounds **68–70** showed carbonic anhydrase inhibitory activity, with IC_50_ values of 19.01, 18.24, and 18.65 μM, respectively. These findings highlight their promising bioactivity and suggest their potential as novel pharmacophores for the treatment of conditions such as glaucoma, epilepsy, and cystic fibrosis [47].

### Cytotoxicity

At a concentration of 10 μg/mL, euchretin A (**11**), euchretin J (**15**), and euchretin M (**18**) exhibited moderate inhibitory effects against the human hepatoma cell line 59 T, with inhibition rates of 52.0%, 86.5%, and 71.5%, respectively. In addition, compound **15** showed an inhibition rate of 53.0% against the human gastric cancer cell line SCM-1 at the same concentration [21]. In a separate study on the same plant, coumaronochromone **50** was reported to inhibit prostate-specific antigen (PSA) secretion with an IC_50_ value of 0.28 μM, while displaying low cytotoxicity [36].

The inflorescences of *Celosia cristata* are used in TCM for hemostasis, as documented in Chinese Pharmacopoeia (2025) [55]. Its constituent, cristatone II (**72**), exhibited significant inhibitory activity against HeLa and BGC-823 cancer cell lines with IC_50_ values of 23.82 and 3.34 μM, respectively [6].

Suaeglaucin C (**77**) exhibited cytotoxicity on HCT116 cells at 30 and 50 μM, and showed marked lethality with a median lethal concentration (LC_50_) of 9.41 μM in healthy zebrafish embryos [52].

### Insecticidal

Obiongin (**38**) exhibited insecticidal activity against the larvae of *Aedes albopictus*, with a LC_50_ value of 5.85 mg/L. In cytotoxicity evaluations, **38** demonstrated potent inhibitory effects against both *Spodoptera litura* (SL) cells and the insect cell lines BTI-TN-5B1-4 (Hi-five). Compound **41** similarly showed high toxicity toward these cell lines, with inhibition rates of 68.41% against SL cells and 56.11% against Hi-five cells. Notably, the cytotoxic activities of both **38** and **41** were significantly higher than those of the positive control rotenone (which exhibited inhibition rates of 20.83% and 21.53% against SL and Hi-five cells, respectively) [32].

### Immunomodulatory

*Campylotropis hirtella*, a shrub widely distributed in subtropical regions of China, has traditionally been used in folk medicine, with its stems and roots employed for the treatment of benign prostatic hyperplasia (BPH). The plant is recognized for its rich profile of flavonoids, indicating a well-developed flavonoid biosynthetic system. Among these, the coumaronochromones hirtellanine A (**49**) and 4',5'-dihydroxy-5,7-dimethoxy-6-(3-methylbut-2-enyl)coumaronochromone (**50**) were isolated from the roots of *C. hirtella*. In vitro studies revealed that compound **49** exhibits potent immunosuppressive activity with IC_50_ values of 0.06 µM against B-lymphocyte proliferation and 0.92 µM against T-lymphocyte proliferation. Cytotoxicity assays using mouse splenic lymphocytes showed that **49** possessed low cytotoxicity, with CC_50_ values of 3.03 µM (B-cells) and 26.32 µM (T-cells), suggesting its potential as a promising lead for novel immunosuppressive agents [8]. Similarly, compound **50** demonstrated significant immunosuppressive activity in mitogen-induced mouse splenocyte proliferation assays, with IC_50_ values of 0.28 µM (B-cells) and 1.55 µM (T-cells), while maintaining low cytotoxicity (CC_50_ = 1.34 µM) [36, 37].

## Construction of the coumaronochromone core

**Scheme 2:**
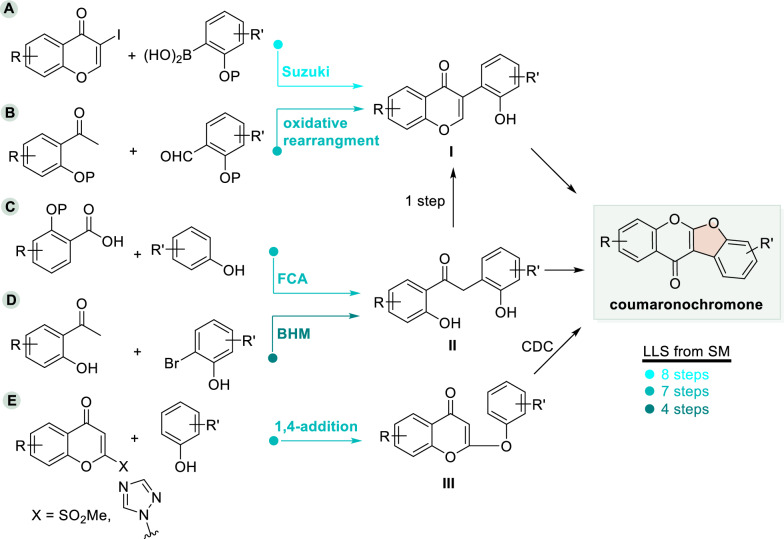
Strategies for construction of the coumaronochromone core

As outlined in Scheme 2, five main strategies have been reported to construct the core scaffold of coumaronochromones. The longest linear sequence from starting materials (SM) to the coumaronochromone skeleton consists of 8 steps, while the shortest requires only 4 steps. These synthetic approaches generally proceed through one of three key intermediates (I–III). Although direct cross-coupling between a chromone C-3 position and an aryl halide may appear efficient [11], this route often limits the late-stage introduction of polyhydroxy and other substituents [56]. Therefore, the following strategies have been developed: **Route A** employs a Suzuki–Miyaura cross-coupling to afford isoflavone **I**, which subsequently undergoes intramolecular oxidative cyclization to form the coumaronochromone core [57, 58]. **Route B** involves oxidative rearrangement of a chalcone to give isoflavone **I**, followed by intramolecular oxidative cyclization to yield the target structure [59]. **Route C** proceeds via Friedel–Crafts acylation of a phenol with a benzoic acid derivative to generate the key α-aryl phenone intermediate **II**. This intermediate is then condensed and cyclized to isoflavone **I**, and finally oxidized to the coumaronochromone [60]. **Route D** utilizes a Buchwald–Hartwig-Miura (BHM)-arylation between an acetophenone and a bromobenzene to produce α-aryl phenome **II**, which is transformed into the coumaronochromone via a tandem condensation/oxidative cyclization sequence [61]. **Route E** relies on nucleophilic addition–elimination between an activated chromone (C-2 position) and a phenol to give the key 2-phenoxychromone intermediate **III**. Intramolecular cross-dehydrogenative coupling (CDC) then delivers the coumaronochromone [48].

### Suzuki–Miyaura cross-coupling enabled total syntheses of lupinalbins A and H

**Scheme 3:**
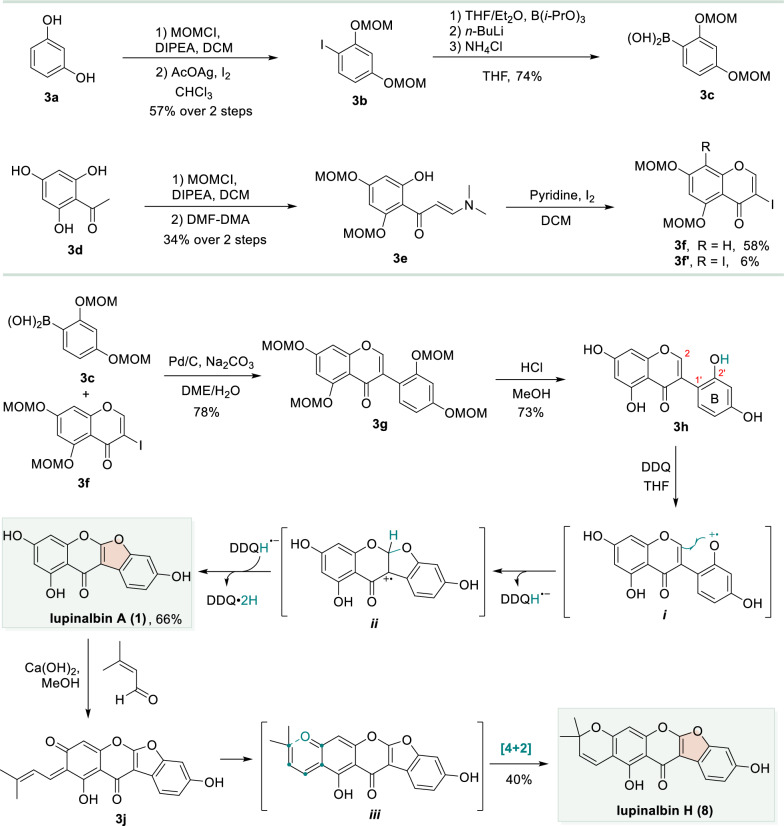
Total syntheses of lupinalbins A and H through Suzuki–Miyaura reaction

Van Heerden et al. used resorcinol **3a** and phloroglucinol **3d** as the starting materials for total syntheses of lupinalbins A and H. Starting from the protection of the phenolic hydroxyl groups of **3a** with MOM chloride, followed by iodination, afforded the iodobenzene **3b**. This intermediate underwent lithium-iodine exchange, nucleophilic substitution with B(O*i*Pr)_3_, and subsequent hydrolysis with NH_4_Cl to give phenylboronic acid **3c**. Concurrently, phloroglucinol **3d** was subjected to MOM protection and then condensed with DMF-DMA to furnish enamine **3e**. Treatment of **3e** with iodine induced cyclization and iodination, providing 3-iodochromone **3f** as the major product, along with a minor amount of iodobenzene by-product **3f′** (Scheme 3) [57]. Subsequently, a Suzuki–Miyaura cross-coupling between **3c** and **3f** then delivered isoflavone **3g**. Deprotection of the MOM protections using HCl/MeOH afforded isoflavone **3h**. Subsequent oxidation with DDQ, proceeding via hydrogen atom transfer (HAT) and single-electron transfer (SET) pathways to generate key intermediates ***i*** and ***ii***, enabled an intramolecular cross-dehydrogenative coupling (CDC) between the 2′-hydroxy group on the B-ring with the C-2 site. This key step successfully furnished the natural product lupinalbin A (**1**). Finally, an aldol reaction between prenal and lupinalbin A (**1**) yielded intermediate **3j**, which spontaneously underwent an intramolecular [4 + 2] cyclization (via transition state ***iii***) to complete the synthesis of the natural product lupinalbin H (**8**) [58].

### Tl(III)-mediated oxidative rearrangement enabled the total synthesis of lupinalbin A and lupilutin

**Scheme 4:**
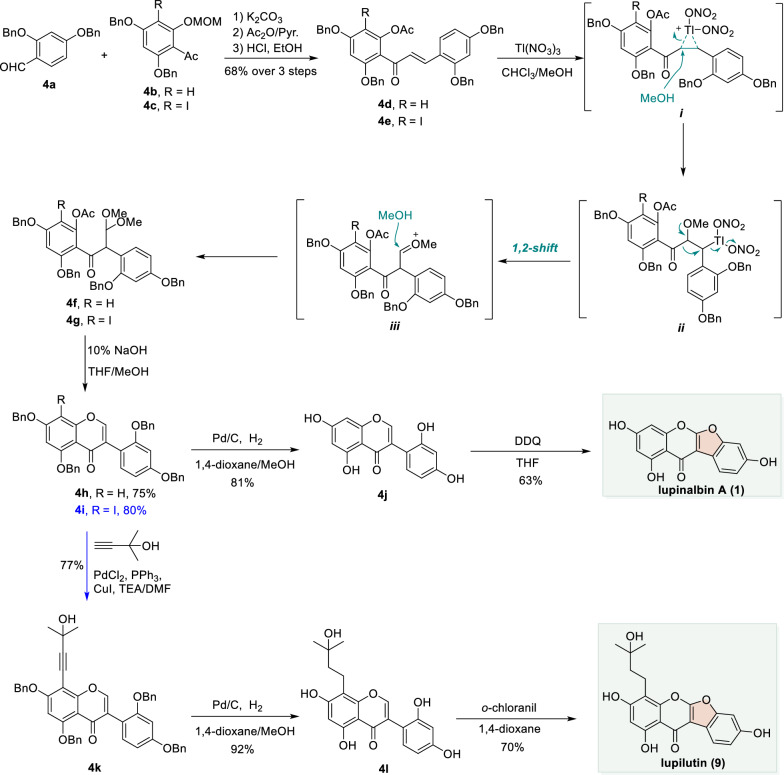
Total syntheses of lupinalbin A and lupilutin through Tl(III)-mediated oxidative rearrangement

Tsukayama and co-workers reported the total syntheses of lupinalbin A and lupilutin starting from functionalized substrates **4a–c**, which were first converted via aldol addition, protection (Ac_2_O), and elimination sequences to furnish chalcones **4d** and **4e** (Scheme 4). These intermediates subsequently underwent a Tl(III)-mediated oxidative rearrangement (via intermediates ***i*–*iii***) to yield acetals **4f** and **4g**. Under basic conditions (10% NaOH/MeOH), **4f** and **4g** then underwent 1,4-addition/elimination to afford isoflavones **4h** and **4i**, respectively. Isoflavone **4h** was subjected to debenzylation using Pd/C under a H_2_ atmosphere to give **4j**, which then underwent a DDQ-mediated oxidative cyclization successfully yielding the natural product lupinalbin A (**1**).

In parallel, isoflavone **4i** was coupled with 2-methyl-3-butyn-2-ol via a Pd-catalyzed Sonogashira cross-coupling reaction to afford **4k**. Subsequent debenzylation (Pd/C, H_2_) furnished isoflavone **4l**. Finally, treatment of **4l** with *o*-chloranil—an oxidant that operates through a similar mechanism to that of DDQ-enabled CDC reaction completed the synthesis of the natural product lupilutin (**9**) (Scheme 4) [59].

### Friedel–Crafts acylation enabled total synthesis of lupinalbin A

**Scheme 5:**
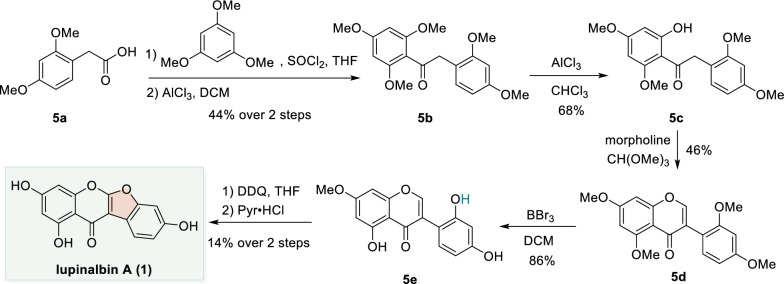
Total synthesis of lupinalbin A through Friedel–Crafts acylation

In the total synthesis of lupinalbin A (**1**) reported by Miller et al., 2,4-dimethoxyphenylacetic acid (**5a**) was condensed with 1,3,5-trimethoxybenzene through Friedel–Crafts acylation to afford the α-aryl phenome **5b**. Selective demethylation of **5b** with AlCl_3_ in chloroform gave the *ortho*-hydroxy phenome **5c**, which subsequently underwent intramolecular cyclization using CH(OMe)_3_ in the presence of morpholine to furnish isoflavone **5d**. Treatment of **5d** with BBr_3_ effected the regioselective demethylation, yielding the 2′-hydroxyisoflavone **5e**. Finally, oxidative cyclization with DDQ followed by global demethylation using pyridinium hydrochloride provided **1** (Scheme 5) [60].

### BHM arylation enabled total synthesis of cristatone II

**Scheme 6:**
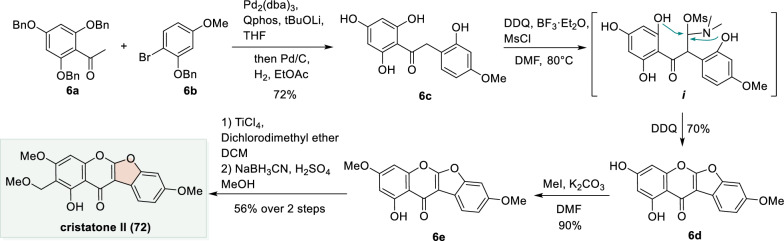
Total synthesis of cristatone II through BHM arylation

The BHM arylation represents an efficient method for aryl group introduction [62–65]. In our laboratory, a de novo synthetic route was developed starting from acetophenone **6a** and bromobenzene **6b** (Scheme 6). A palladium-catalyzed intermolecular BHM arylation afforded the corresponding α-aryl phenone, which after debenzylation (Pd/C, H_2_) yielded α-aryl phenome **6c**. Subsequently, **6c** was treated with activated DMF under the conditions of DDQ/BF_3_·OEt_2_/MsCl to generate the reactive intermediate ***i***. In the presence of DDQ, intermediate ***i*** underwent a tandem oxidative cyclization to furnish coumaronochromone **6d**. Selective methylation of **6d** (MeI, K_2_CO_3_) then provided **6e**. Finally, application of a modified Vilsmeier–Haack reaction (TiCl_4_, dichlorodimethyl ether) followed by selective reduction and methylation (NaBH_3_CN/H_2_SO_4_/MeOH) completed the total synthesis of the natural product cristatone II (**72**) [61].

### Chromone-based 1,4-addition/elimination enabled total synthesis of lupinalbin A

**Scheme 7:**
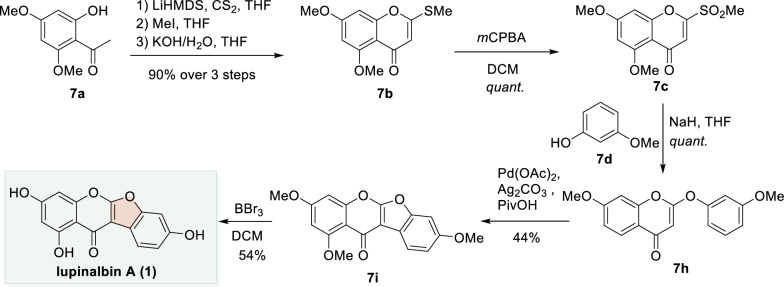
Total synthesis of lupinalbin A through a chromone-based 1,4-addition/elimination strategy

In the synthetic route developed by Xu and Chen et al., 2-hydroxyacetophenone **7a** was converted to 2-thiochromone **7b** via a three-step sequence involving nucleophilic addition to CS_2_, cyclization, and elimination. Subsequent oxidation of **7b** with *m*CPBA furnished the activated 2-mesylchromone **7c**. Treatment of **7c** with the phenol derivative **7d** in the presence of NaH promoted a 1,4-addition/elimination process, affording the 2-phenoxychromone **7h**. Notably, **7h** could also be prepared from a corresponding 3-triazolochromone via an analogous transformation. Palladium-catalyzed CDC reaction of **7h** then delivered the coumaronochromone scaffold **7i**. Final demethylation with BBr_3_ completed the synthesis of the natural product lupinalbin A (**1**) (Scheme 7) [48, 66, 67].

## Conclusion

Coumaronochromones represent a significant subclass of natural isoflavonoids. A comprehensive review summarizing their natural occurrence, botanical origins, biosynthetic pathways, pharmacological activities, and chemical synthesis strategies will provide valuable insights and facilitate further research and development of these natural products. These compounds are primarily distributed in plants of the Fabaceae and Moraceae families. Notably, some source plants, such as *Euchresta formosana* and *Desmodium styracifolium*, have a long history of use in TCM, underscoring their therapeutic relevance. Corresponding coumaronochromones exhibit a broad spectrum of bioactivities, including anti-inflammatory, antibacterial, antitumor, and immunomodulatory effects, highlighting their considerable potential in drug discovery. In addition, the chemical synthesis of coumaronochromones has been achieved through diverse strategies. Conventional approaches for constructing the core benzofuro[2,3-*b*]chromenone scaffold include Suzuki–Miyaura cross-coupling, Tl-mediated oxidative rearrangement, Friedel–Crafts acylation, chromone-based 1,4-addition/elimination, and subsequent DDQ-mediated cross-dehydrogenative coupling (CDC) cyclization. Recently, a novel modular strategy featuring a BHM arylation/DDQ-oxidative cyclization sequence was developed. This efficient four-step protocol offers mild reaction conditions and excellent functional-group tolerance, and has been successfully applied to the concise seven-step total synthesis of the natural product cristatone II. With the rapid advancement of green chemistry and synthetic biology, more efficient and environmentally benign synthetic methodologies for this promising class of compounds are anticipated to emerge.

## Data Availability

The data availability is not applicable.
